# Introduction and Dispersal of Sindbis Virus from Central Africa to Europe

**DOI:** 10.1128/JVI.00620-19

**Published:** 2019-07-30

**Authors:** Jiaxin Ling, Teemu Smura, Jan O. Lundström, John H.-O. Pettersson, Tarja Sironen, Olli Vapalahti, Åke Lundkvist, Jenny C. Hesson

**Affiliations:** a Zoonosis Science Center, Department of Medical Biochemistry and Microbiology, Uppsala University, Uppsala, Sweden; b Department of Virology, Medicum, University of Helsinki and Helsinki University, Helsinki, Finland; c Biologisk Myggkontroll, Nedre Dalälven Utvecklings AB, Uppsala, Sweden; d Marie Bashir Institute for Infectious Diseases and Biosecurity, Charles Perkins Centre, School of Life and Environmental Sciences, University of Sydney, Sydney, New South Wales, Australia; e Sydney Medical School, University of Sydney, Sydney, New South Wales, Australia; f Public Health Agency of Sweden, Solna, Sweden; g Department of Veterinary Biosciences, University of Helsinki, Helsinki, Finland; University of North Carolina at Chapel Hill

**Keywords:** evolution, phylogeny, Sindbis virus

## Abstract

Bird-hosted viruses have the potential to be transported over large areas of the world and to be transmitted in distant geographical regions. Sindbis virus (SINV) is a mosquito-borne alphavirus that is locally amplified in a bird-mosquito enzootic cycle and distributed all over the Old World and Australia/Oceania. Sindbis virus genotype I (SINV-I) is the cause of disease outbreaks in humans in South Africa as well as in northern Europe. To trace the evolutionary history and potential strain-disease association of SINV-I, we sequenced 36 complete genomes isolated from field material in Europe, as well as in Africa and the Middle East, collected over 58 years. These were analyzed together with 30 additional published whole SINV-I genomes using Bayesian analysis. Our results suggested that SINV-I was introduced only once to northern Europe from central Africa, in the 1920s. After its first introduction to Sweden, it spread east and southward on two separate occasions in the 1960s and 1970s. Another introduction from central Africa to southern/central Europe seems to have occurred, and where these two introductions meet, one recombination event was detected in central Europe. In addition, another recombinant strain was found in central Africa, where the most divergent SINV-I strains also originated.

**IMPORTANCE** This study shows that only a single introduction of SINV into a new geographical area is required for spread and establishment, provided that the requisite vector(s) and reservoir(s) of epizootological and epidemiological importance are present. Furthermore, we present the first report of recombination between two strains of SINV in nature. Our study increases the knowledge on new introductions and dispersal of arboviruses in general and of SINV in particular.

## Abstract (precis)

This study shows that only a single introduction of SINV into a new geographical area is required for spread and establishment, provided that the requisite vector(s) and reservoir(s) of epizootological and epidemiological importance are present. Furthermore, we present the first report of recombination between two strains of SINV in nature. Our study increases the knowledge on new introductions and dispersal of arboviruses in general and of SINV in particular.

## INTRODUCTION

Viruses hosted by birds have the potential to be transported over large areas of the world, connecting countries and regions that are well separated in climate and habitat. The occurrence of human disease from vector-borne bird viruses is dependent on the availability and abundance of competent vectors in proximity to the bird hosts and humans in different regions. Thus, these viruses need to be able to replicate in various vector and host species, as the geographic distribution often spans tropical as well as subtropical or temperate regions, with unique sets of mosquito and bird species.

Mosquito-borne bird viruses include a number of alphaviruses (*Togaviridae*), e.g., Sindbis virus (SINV) and Western equine encephalitis virus (WEEV), and flaviviruses (*Flaviviridae*), e.g., West Nile virus (WNV), Japanese encephalitis virus (JEV), Murray Valley encephalitis virus (MVEV), St. Louis encephalitis virus (SLEV), Rocio virus (ROCV), and Usutu virus (USUV) (1–5). In addition to their dependence on avian hosts, all these viruses also utilize vectors from the same genus of mosquitoes, *Culex*. This genus has species representatives throughout the world; thus, it has the potential to provide suitable vector species in both tropical and temperate regions (2, 6–8). Human cases emerge from an infectious mosquito bite and are considered an effect of spillover from a viremic bird population. The viremia produced in humans is not enough to infect a mosquito, and consequently, humans are dead-end hosts of the virus (2).

SINV is a positive single-stranded RNA virus that has a wide distribution throughout the Old World. It has a genome size of about 11.7 kb and has two open reading frames (ORFs) that encode four nonstructural (nsP1 to -4) and five structural (C, E3, E2, 6K/TF, and E1) proteins, respectively (9). Based on phylogenetic analyses of the partial E2 gene, a total of six genotypes (genotypes I to VI) have been identified (10). SINV genotype I (SINV-I) has been isolated from Europe, Africa, and the Middle East; SINV-II and SINV-VI have been isolated from Australia; SINV-III has been isolated from Southeast Asia; SINV-IV has been isolated from Asia and the Middle East; and SINV-V (also referred to as Whataroa virus) has been isolated from New Zealand (10).

SINV-I is the only genotype that has been associated with outbreaks of human disease, and it was first isolated from mosquitoes in Cairo, Egypt, in 1952 (11). Outbreaks have been reported from South Africa and northern Europe, and the disease goes under several names: Pogosta, Ockelbo disease, Karelian fever, and Sindbis fever. Signs and symptoms include fever, exanthema, arthralgia, myalgia, and cases of arthritis that can remain for years (12, 13).

The first cases of Sindbis fever in Fennoscandia were reported from Sweden in the 1960s, and later, this disease was shown to be associated with a strain of SINV-I isolated from mosquitoes in Edsbyn, Sweden (14). Cases of Sindbis fever are sporadically diagnosed in central Sweden (15), and in recent years, there has also been an outbreak of Sindbis fever in northern Sweden (16). Several larger outbreaks have been observed in Finland and in South Africa (17, 18). However, as mentioned above, SINV-I has also been isolated from other parts of Africa, Europe, and the Middle East, without any reports of disease outbreaks. Previous studies indicated that SINV-I has been transported with northward-migrating birds, connecting South Africa and Fennoscandia (10). The driving forces behind SINV-I outbreaks and the details of SINV-I movements between continents and regions are still unknown.

To further elucidate the evolutionary history of SINV-I and its dispersal patterns, we have sequenced and analyzed the complete genomes of 36 new strains, in addition to 30 previously sequenced publicly available strains, from various vector and host species, spanning 10 countries and 58 years.

## RESULTS

### Evolutionary history of SINV-I.

**FIG 1:**
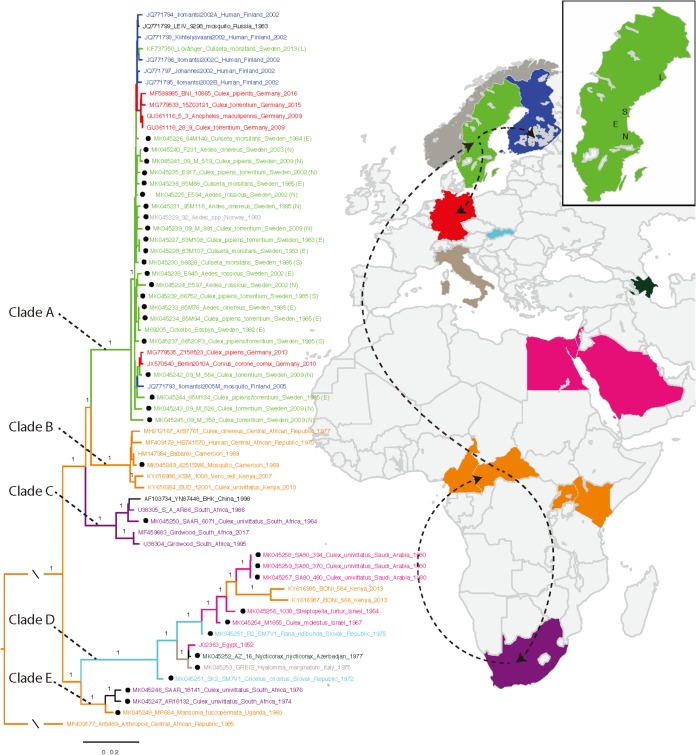
Spread of SINV-I from Africa to Europe. Shown is a Bayesian phylogenetic tree of SINV-I based on concatenated nucleotide ORF sequences (MrBayes). The colors indicate Sweden (green); Finland (blue); Germany (red); Egypt, Israel, and Saudi Arabia (pink); Kenya and the Central African Republic (orange); Slovak Republic (light blue); Italy (gray); and South Africa (purple). The roots in orange indicate that SINV-I originated in central Africa. Main SINV-I dispersal routes from Africa to and within northern/central Europe are indicated with dashed lines. L, N, E, and S represent locations in Sweden (Lövånger, Nedre Dalälven, Edsbyn, and Sundsvall, respectively). Posterior probability values above 0.95 are shown for the branches. Dots indicate the sequences generated from this study.

The phylogenetic tree of the complete coding sequence of SINV-I contained 64 strains (2 strains were excluded due to recombination [see below]) and showed that the strains from central Africa were basal in each clade. According to the parsimony principle (i.e., that the best hypothesis is the one that requires the fewest evolutionary changes), this indicates that SINV-I originated in central Africa and diverged into two main clades with well-supported values (posterior probability, 1.0) (Fig. 1). Clade A contained all the strains from Sweden, Finland, Norway, Germany, and Russia and shared a common ancestor with strains from clade B, which included strains from central African countries (i.e., Central African Republic, Cameroon, and Kenya). Clades A and B shared a common ancestor with the strains from South Africa (clade C). The other main clade (clade D) contained the prototype SINV-I strain from Egypt (1952), which clustered with sequences from other countries in the Middle East and adjacent regions (i.e., Egypt, Israel, Saudi Arabia, and Azerbaijan), southern/central Europe (Slovak Republic and Italy), and east Africa (Kenya). Clade D shared a most recent common ancestor with clade E, which included strains from central Africa (Uganda) and South Africa. However, these two clades (clades D and E) contained only 15 sequences, including some from unconventional hosts, and therefore, a more detailed analysis of this group could not be made.

**FIG 2:**
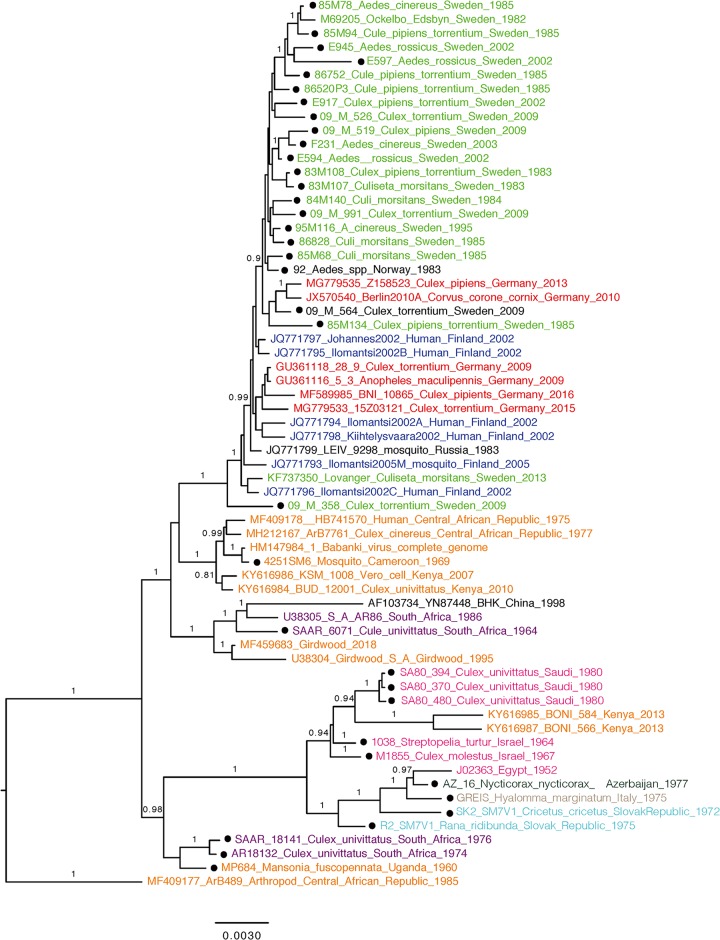
Bayesian phylogenetic tree of SINV-I based on the complete open reading frame (ORF) amino acid sequences resulting from MrBayes. Dots indicate the sequences generated from this study. Posterior probability values above 0.8 are shown for the branches.

The most parsimonious interpretation is that SINV-I strains circulating in central and northern Europe were introduced from central Africa rather than from South Africa, as previously hypothesized (10). Analysis of clade A, which contains most European strains, shows that the basal position was held by a strain from Nedre Dalälven, Sweden (09_M_358; GenBank accession number MK045245), suggesting that SINV-I was first introduced from central Africa to Sweden, with subsequent circulation there, and then further dispersed from Sweden to other parts of northern, eastern, and central Europe (Fig. 1). This was further confirmed by the phylogenetic tree based on amino acid sequences of SINV-I, which had the same topology (Fig. 2).

### Evolutionary rates.

**FIG 3:**
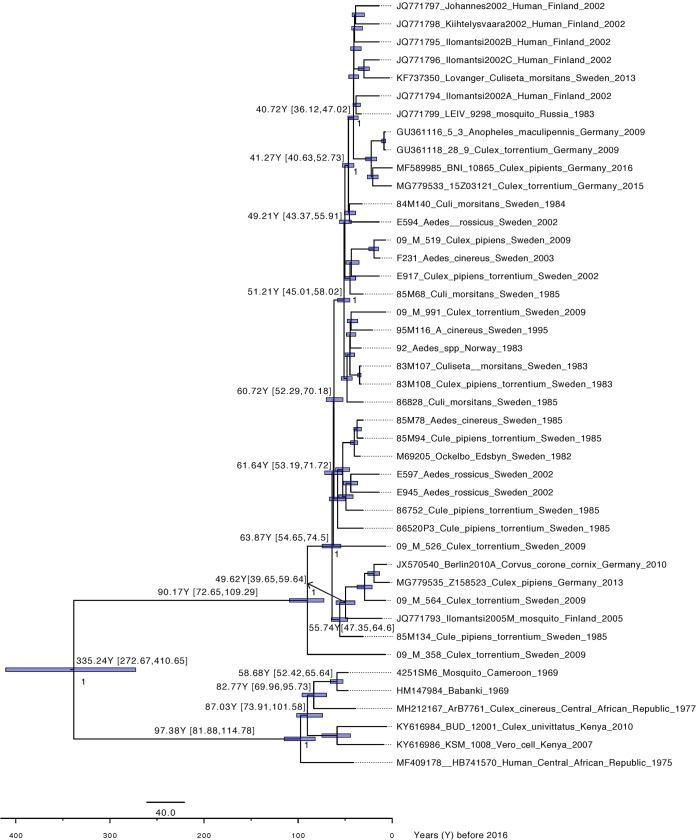
Dated phylogenetic tree of clades A and B. Posterior probability values above 0.95 are shown in the nodes. The values on the branches indicate years before 2016 and 95% highest posterior density (HPD) values.

A TempEst analysis showed that the whole data set (64 sequences) did not have any temporal structure (correlation coefficient, −0.0904). However, the data set including sequences from clades A and B showed a temporal signal (43 sequences; correlation coefficient, 0.5128) and was subsequently used to estimate the evolutionary rate of clades A and B. Following model testing, the evolutionary history of clades A and B of SINV-I was reconstructed with a strict molecular clock mode and a coalescent exponential population demographic model. The evolutionary rate for this data set is 5.45 × 10^−5^ substitutions (95% highest posterior density [HPD], 4.40 × 10^−5^ to 6.56 × 10^−5^) (Table 1). The time to the most recent common ancestor (tMRCA) estimated that clade A dated back to 93 years ago (95% HPD, 76 to 112 years), indicating that SINV-I was introduced into Sweden in the 1920s. This was then followed by two separate introductions of SINV-I from Sweden into Finland and Germany around the 1960s and the 1970s, respectively, which corresponds to epidemiological data on SINV infection in northern Europe (Fig. 3). Notably, we also estimate that clade A and clade B diverged from their African ancestors more than 300 years ago (95% HPD, 1605 to 1743) and perhaps longer if the data have been impacted by strong purifying selection (19). Hence, prior to the emergence of SINV-I in northern Europe, there were more than 300 years of viral diversity and spread that are still unaccounted for.

**TABLE 1:** Identities of nucleotide and amino acid sequences of SINV-I strains within different clades

Identity category^*a*^	% identity									
	CDS	nsP1	nsP2	nsP3	nsP4	CP	E3	E2	6K	E1
Total (nt)	87.9–100	91.8–100	85.8–100	84.4–100	88–100	88.7–100	86.6–100	87.6–99.9	88.1–100	89.1–100
Total (aa)	96.3–100	97.1–100	96.9–100	91.1–100	97.5–100	92.2–100	94.5–100	95.3–100	94.2–100	96.2–100
Within clade A (nt)	99.1–100	99.2–100	98.9–100	99–100	98.9–100	96.8–100	98.8–100	99–99.9	97.5–100	99–100
Within clade A (aa)	99.1–100	98.9–100	99.3–100	89.3–100	99.3–100	94.4–100	98.2–100	99–100	96.2–98.1	99.3–100
Within clade B (nt)	99.1–99.9	99.1–99.8	99.1–99.9	99.3–99.9	99.1–100	98.8–100	99.4–100	99–99.8	98.8–100	99.1–99.9
Within clade B (aa)	99.7–100	99.3–99.8	99.8–100	99.6–100	99.7–100	99.2–100	100	99.8–100	100	99.1–100
Within clade C (nt)	98.3–99.8	98.6–99.9	98.1–99.6	97.6–99.6	98.7–99.7	98.7–100	97.6–100	98.3–99.8	95.4–100	98.4–100
Within clade C (aa)	98.9–99.6	98.5–100	98.5–99.6	97.6–99.5	98.9–100	99.2–100	96.4–100	98.8–100	96.2–100	98.6–100
Within clade D (nt)	87.9–100	96.1–100	94.3–100	95.7–100	96.1–100	95.7–100	97–100	94.9–100	93.2–100	97–100
Within clade D (aa)	96.3–100	97.9–100	97.7–100	96.6–100	98–100	97.7–100	100	97.3–100	98.1–100	98.6–100
Within clade E (nt)	99.2–99.6	99.4–99.7	98.9–99.8	98.5–99.3	99.3–99.4	99.1–99.7	98.8–99.4	99.3–99.8	93.1–98.8	99.3–100
Within clade E (aa)	99.6–99.9	99.8–100	99.8–100	99–99.6	99.8–100	99.2–100	100	99.8–100	94.2–98.1	94.8–100

a nt, nucleotides; aa, amino acids.

### Phylogeny of the partial E2 gene.

**FIG 4:**
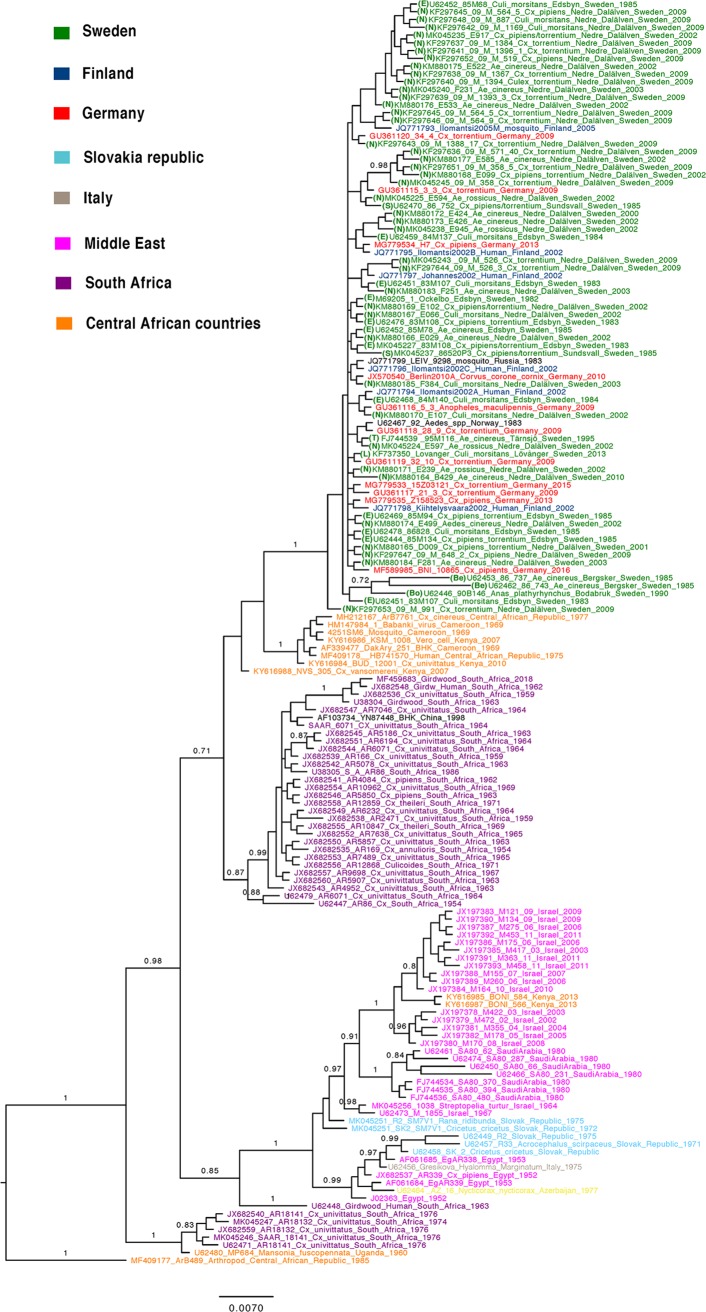
Bayesian phylogenetic tree of SINV-I based on partial E2 sequences. Posterior probability values above 0.8 are shown for the branches.

We also performed the same analysis on 163 sequences of the partial E2 alignment (Fig. 4), and the results showed that it could not discriminate any additional within-clade geographical structure compared to the analysis of the complete ORFs. Northern/eastern SINV strains also clustered together in the partial E2 phylogeny. However, it failed to detect the origin of this clade, as both the central African and South African clades were mixed together, and the overall posterior probability support was low.

### Genetic diversity of SINV-I.

The SINV-I genome was highly conserved, with an average nucleotide similarity of 96.5% (range, 87.9% to 100%). The similarity of the nonstructural protein genes nsP1 (97.4%; range, 91.8% to 100%), nsP2 (96.3%; range, 85.8% to 100%), nsP3 (95.4%; range, 84.4% to 100%), and nsP4 (96.4%; range, 88% to 100%) showed similar levels compared to the structural protein genes capsid (96.7%; range, 88.7% to 100%), E3 (96.7%; range, 86.6% to 100%), E2 (96.2%; range, 87.6% to 99.9%), 6K/TF (96.8%; range, 88.1% to 100%), and E1 (97.5%; range, 89.1% to 100%), with an average nucleotide similarity ranging from 95.4% (nsP3) to 97.5% (E1).

**FIG 5:**
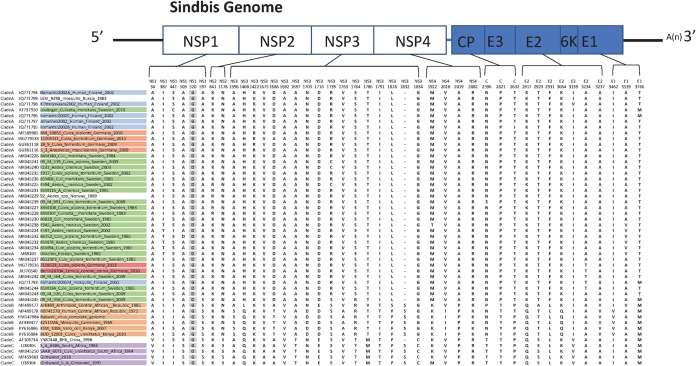
Comparison of amino acid variations across the SINV-I genome among the strains in clades A, B, and C. The colors of the strains indicate Sweden (green), Finland (blue), Germany (red), Kenya and the Central African Republic (orange), and South Africa (purple).

A comparison of nucleotide and amino acid differences showed that the highest degree of observed divergence was found in clade D (Table 1). Within clades A, B, and C, where most strains from regions with reported outbreaks were located, no specific differences could be observed that clearly separated strains isolated from human patients from other strains (Fig. 5). Thus, direct disease association could not be accounted for by any unique amino acid substitution.

### Recombination within SINV-I.

**FIG 6:**
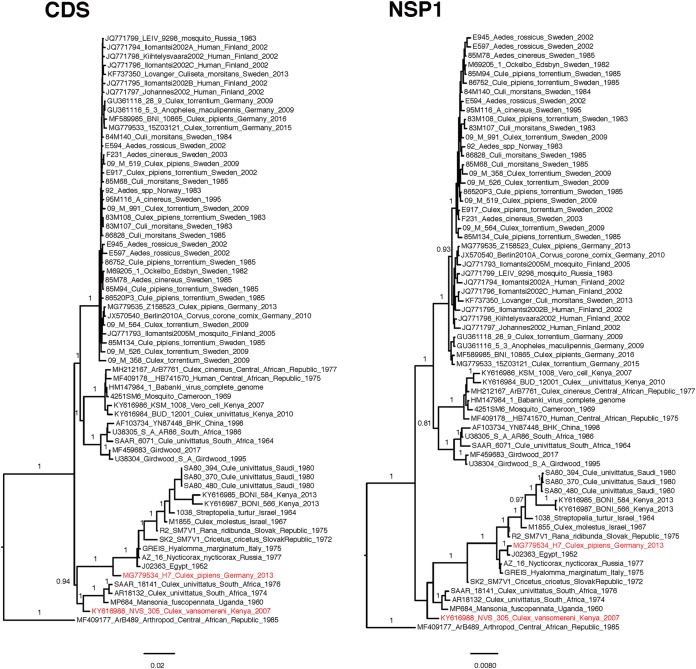

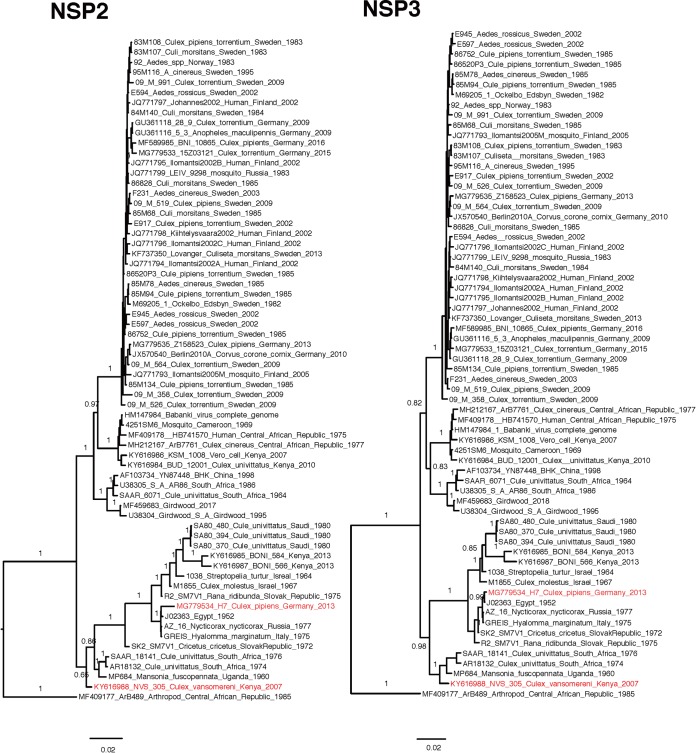

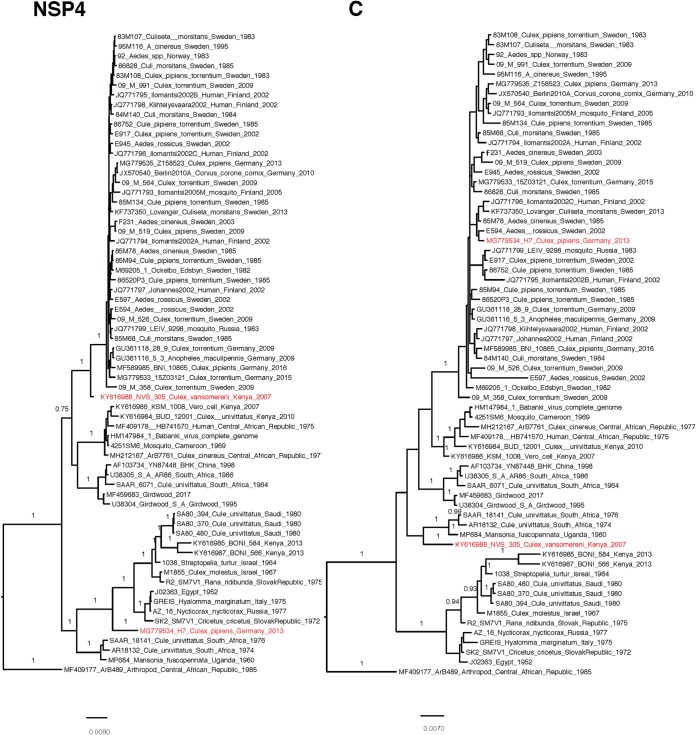

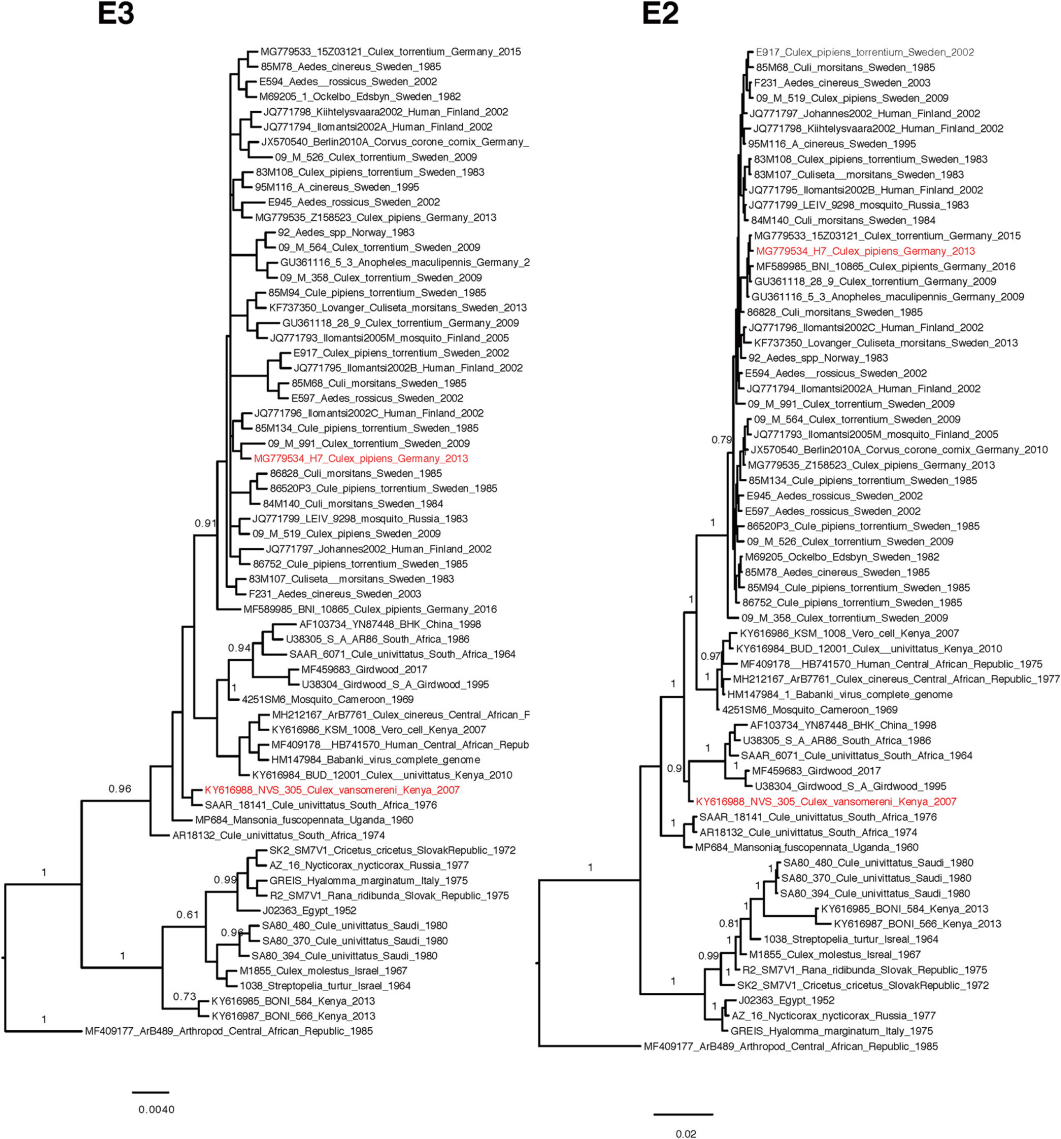

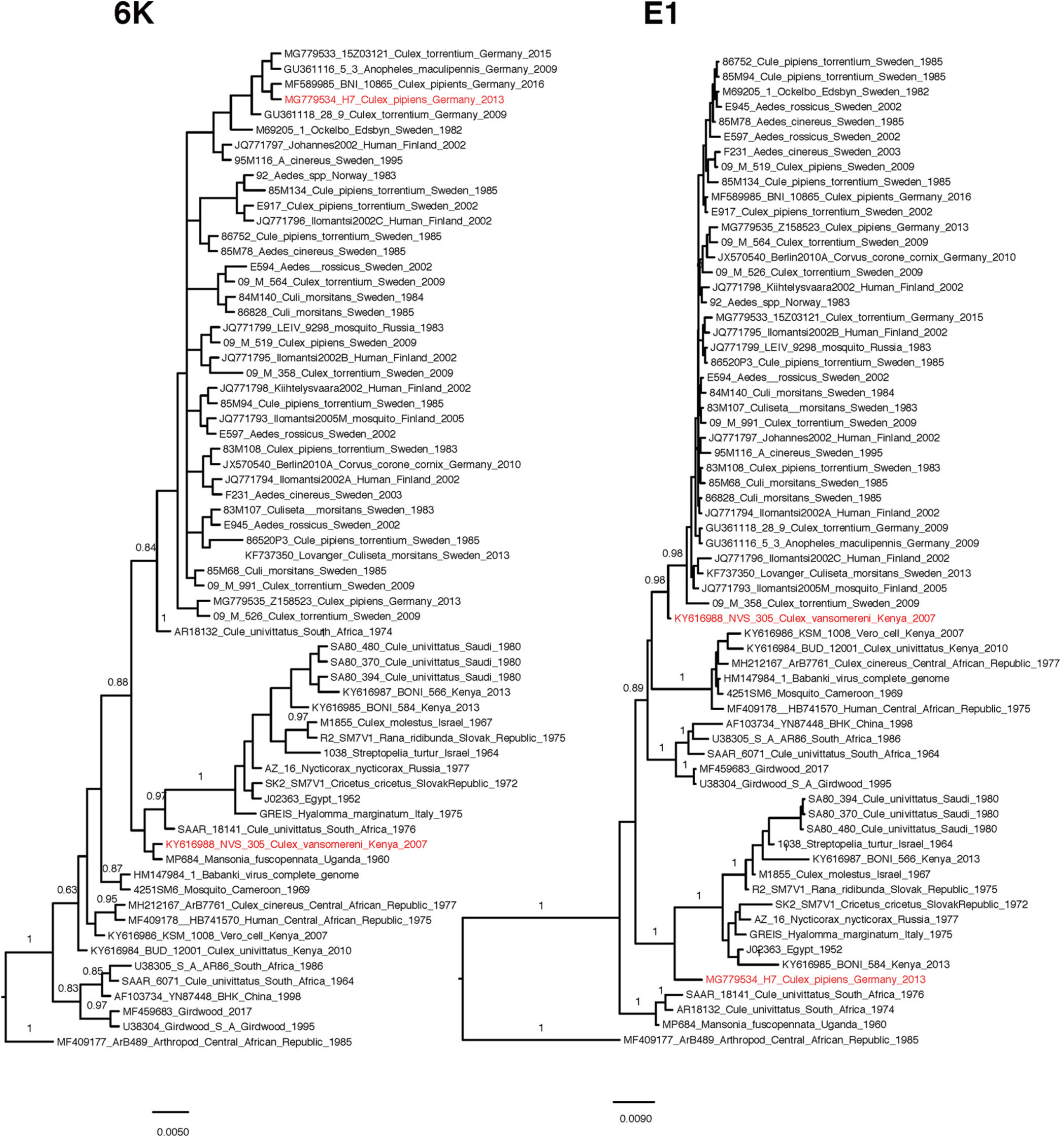
Bayesian phylogenetic trees based on the complete ORFs and the separated genes (nsP1 to -4, C, E3, E2, 6K/TF, and E1) of fully sequenced SINV-I genomes. Sequences that have undergone recombination are highlighted in red. CDS, coding sequence.

**FIG 7:**
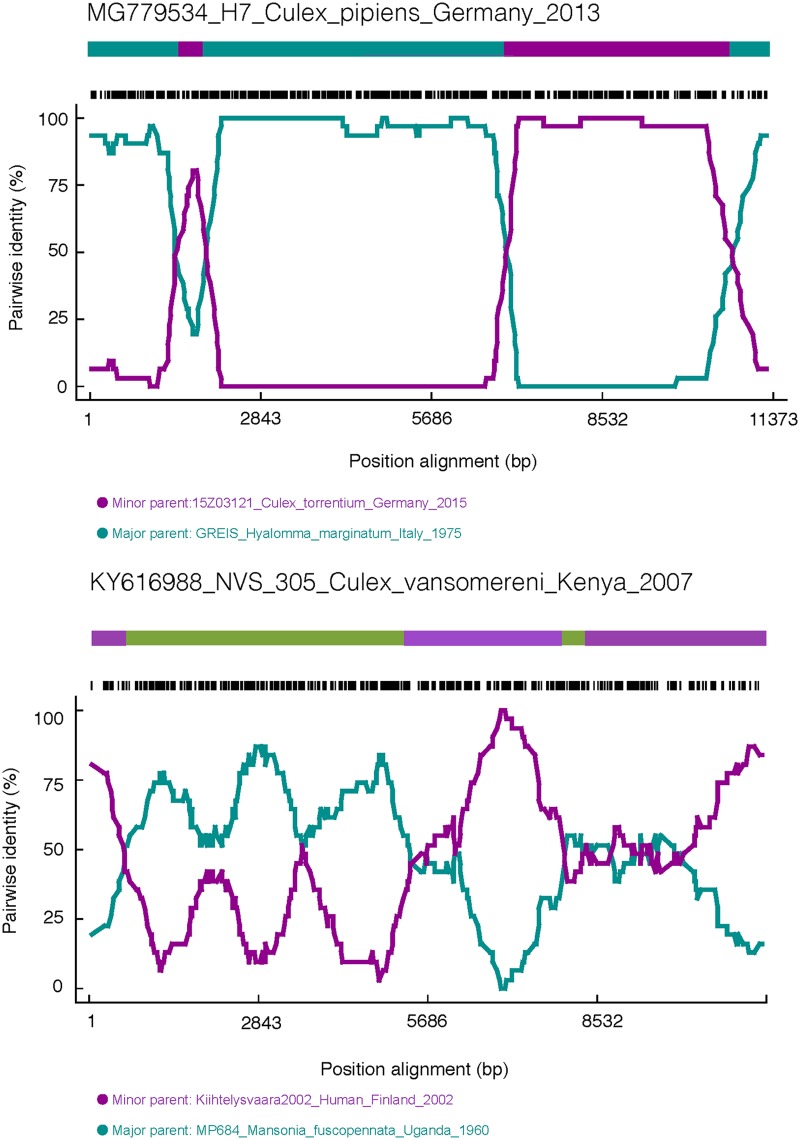
Recombination scheme for strains H7_Culex_pipiens_Germany_2013 and NVS_305_Culex_vansomereni_Kenya_2007. The recombination events were checked by using RDP in RDP3, and the window size (the number of polymorphic sites per window) was set to 30.

Two recombination events in the alignment of the complete ORFs were found, showing that the strains NVS_305_Culex_vansomereni_Kenya_2007 (GenBank accession number KY616988) and H7_Culex_pipiens_Germany_2013 (GenBank accession number MG779534) have undergone recombination (Fig. 6). Interestingly, the phylogenies of nsP4 and E1 for NVS_305_Culex_vansomereni_Kenya_2007 were incongruous with those of other genes. Based on nsP4 and E1, this strain clustered in clade A together with the strains from northern Europe, whereas based on the other genes, NVS_305_Culex_vansomereni_Kenya_2007 is a member of clade E. For H7_Culex_pipiens_Germany_2013, incongruences of phylogenies were largely based on structural versus nonstructural protein-coding genes. This strain clustered in clade D on the basis of nonstructural genes and the (structural) E1 gene, whereas the other structural genes (C, E3, E2, and 6K/TF) of this strain were found to be more related to the strains from clade A. This recombinant strain indicates a second introduction of SINV-I to Germany, arriving from the south, which recombined with the strain introduced from the north. The potential breakpoints for these two strains were also confirmed by several methods in RDP4 (Table 2 and Fig. 7).

**TABLE 2:** Recombination analysis for strains H7_Culex_pipiens_Germany_2013 and NVS_305_Culex_vansomereni_Kenya_2007^*a*^

Recombination strain	Event	Minor parental sequence (breakpoint positions [bp])	Major parental sequence	*P* value determined by detection method								
				RDP	GENECONV	Bootscan	Maxchi	Chimera	SiSscan	Phylpro	LARD	3Seq
H7_Culex_pipiens_Germany_2013	1	15Z03121_Culex_torrentium_Germany_2015 (6897–10655)	GREIS_Hyalomma_marginatum_Italy_1975	1.07E−78	2.76E−70	2.46E−76	1.35E−33	8.76E−34	4.41E−39	NS	NS	3.43E−12
		E594_Aedes__rossicus_Sweden_2002	SK2_SM7V1_Cricetus_cricetus_SlovakRepublic_1972									
		84M140_Culi_morsitans_Sweden_1984	J02363_Egypt_1952									
		83M108_Culex_pipiens_torrentium_Sweden_1983	AZ_16_Nycticorax_nycticorax_Russia_1977									
	2	15Z03121_Culex_torrentium_Germany_2015 (1512–1930)	AZ_16_Nycticorax_nycticorax_Russia_1977	1.35E−23	2.90E−22	6.01E−20	1.86E−05	1.83E−05	4.61E−05	NS	NS	3.43E−12
		E594_Aedes_rossicus_Sweden_2002	SK2_SM7V1_Cricetus_cricetus_SlovakRepublic_a_1972									
		84M140_Culi_morsitans_Sweden_1984	GREIS_Hyalomma_marginatum_Italy_1975									

NVS_305_Culex_vansomereni_Kenya_2007	1	Kiihtelysvaara2002_Human_Finland_2002 (5258–7890)	MP684_Mansonia_fuscopennata_Uganda_1960	7.77E−06	2.56E−06	0.001478066	1.63E−07	0.034855155	1.21E−10	NS	NS	8.71E−09
		E594_Aedes__rossicus_Sweden_2002	SAAR_18141_Cule_univittatus_South_Afria_1976									
		84M140_Culi_morsitans_Sweden_1984	Culex_univittatus_South_Africa_1974									
	2	JQ771793_Ilomantsi2005M_mosquito_Finland_2005 (8274–596)	SAAR_18141_Cule_univittatus_South_Afria_1976	4.45E−06	5.18E−05	0.000194763	2.12E−06	2.27E−06	3.71E−06	NS	NS	0.010431756
		E594_Aedes_rossicus_Sweden_2002	Culex_univittatus_South_Africa_1974									
		84M140_Culi_morsitans_Sweden_1984	MP684_Mansonia_fuscopennata_Uganda_1960									

a The minor parent is the parent contributing the smaller fraction of sequence. The major parent is the parent contributing the larger fraction of sequence. Breakpoint positions (in base pairs) are relative to E594_Aedes_rossicus_Sweden_2002. NS indicates that no significant *P* value was recorded for this recombination event using this method.

## DISCUSSION

In this study, we have investigated the evolutionary history and dispersal pattern of SINV-I, by phylogenetic analyses of 66 full-genome sequences isolated from regions all throughout its geographical range. The current long-standing hypothesis has been that SINV-I was introduced to northern Europe from South Africa by migratory birds (20–22). This has also been supported by reports of a similar disease occurring in these two regions (12, 18). However, our analyses suggest that the most likely origin of all strains isolated in northern Europe is a single introduction of SINV-I into Sweden from central Africa rather than from South Africa. The specific region from which the virus was exported is, however, uncertain, due to the few and geographically limited isolates of SINV available from Africa. In addition, our results indicate that SINV-I strains were further exported from Sweden to Finland, Russia, and Germany, presenting an eastward and southward dispersal, in contrast to the more commonly proposed northward dispersal of pathogens (23).

The intercontinental dispersals of several bird-hosted viruses, such as WNV and USUV, have been linked to the northward movement of migratory birds (1, 24). In the present study, there is no indication of multiple introductions of SINV-I to northern Europe; thus, northward transport with migratory birds is likely to be a very rare event. This supports conclusions from previous studies on SINV-I phylogeny and the low SINV-I antibody prevalence in northward-migrating birds (20, 25). However, the composition of clade D, containing Middle Eastern and southern/central European strains, implied that southern/central Europe might have had three introductions of SINV-I, two from northern Europe and one from central Africa.

The southward dispersal of SINV-I could be explained by the autumn migration of the main amplifying host species. The enzootic circulation of SINV-I occurs mainly in August, with thrushes of the genus *Turdus* as the main amplifying host of SINV-I in Sweden (15, 21). These *Turdus* species breed in Sweden and migrate southward in August to October to spend the winter in central and southwestern Europe (26). The SINV-neutralizing antibody prevalence in redwing (Turdus iliacus), songthrush (Turdus philomelos), and fieldfare (Turdus pilaris) sometimes exceeds 70% in Sweden (27), which indicates that a substantial number of migrating thrushes are likely to include viremic individuals dispersing SINV-I to local mosquito populations on their way south.

The first report of Sindbis fever in humans in Fennoscandia was from Sweden in 1967 (28). However, prior to this report, there were already signs indicating the existence of SINV in Europe: specific anti-SINV antibodies were found in human sera in northern Italy and Finland already in 1965 (29, 30). Antibody screenings of 5,000 human serum samples in Finland in 1958 to 1964 and in Austria before 1963 (31), however, reported that no antibodies were present. Our dating analysis showed that SINV-I was introduced into Sweden in the 1920s and spread east and southward on two separate occasions to Finland and Germany in the 1960s and 1970s. This is well in agreement with epidemiological data from Finland but also indicates that SINV-I was circulating undetected in Sweden for many decades before it was reported to cause disease. After the confirmation of the first SINV human case in Sweden, sporadic cases as well as recurrent outbreaks have been reported annually in Sweden (32) and Finland (33, 34), as have occasional outbreaks of hundreds or even thousands of human cases. In Finland, SINV-I has been a notifiable disease since 1995, and numbers of reported cases are 10-fold higher than in Sweden, where the awareness of Sindbis fever is considerably lower. Further investigations are needed to clarify if this difference is due to underreporting in Sweden or if it reflects real differences in Sindbis fever occurrence. In both countries, however, the introduction of SINV-I was a rare one-time event, and the virus successfully managed to become established in an enzootic cycle locally, with occasional cases of spillover to humans.

Previous studies have used the E2 gene as a proxy for genotyping SINV-I strains (10). This study confirms that it is a good marker for genotyping; however, it has a limited ability to resolve detailed dispersal patterns. Reconstructing the phylogenies based on separate genes also did not improve the resolution of the phylogeny, despite many more sequences being used in the partial E2 phylogeny. Thus, the best-resolved phylogeny is by using concatenated ORF sequences.

We detected two strains (NVS_305_Culex_vansomereni_Kenya_2007 and H7_Culex_pipiens_Germany_2013) that most likely underwent a recombination event. This is, to our knowledge, the first time that a recombination event has been detected for SINV-I in nature. A previous recombination event between a Sindbis-like virus and Eastern equine encephalomyelitis virus (EEEV) in South America has had a large impact on the alphavirus genus, giving rise to WEEV, Highlands J virus, and Fort Morgan virus (35, 36). The ancestral recombinant obtained E1 and E2 proteins from the Sindbis-like virus and the remaining genes from EEEV (35). The three recombinants, and their variants (e.g., Buggy Creek virus, a variant of Fort Morgan virus), are all vector-borne viruses that infect birds in the New World, but only WEEV has a clear association with human disease.

SINV-I, as for all alphaviruses, evolves relatively slowly (5), which is indicated by the comparatively low genetic diversity observed within the clades and by the finding that model selection favored the constant clock model in this study. Our results showed few genetic differences within SINV-I, especially within the clade of northern European strains (99.1 to 100% overall similarity). The E2 gene encodes a glycoprotein that is associated with the pathogenicity of SINV infections (37, 38). Single substitutions can cause considerable differences in arbovirus vector specificity and pathogenicity, as shown for chikungunya virus (CHIKV) and Zika virus (39, 40). However, we could not detect any clear nucleotide, or any amino acid, differences between SINV-I strains from regions with disease and those without disease (Fig. 5). The reasons for the lack of reported disease cases can be many. In central Africa, several severe diseases are endemic, and underdetection and underreporting would be highly likely. An alternative explanation could be that various ecological factors have an impact on the efficiency of disease transmission, such as a lack of competent and abundant vectors. When considering the lack of human cases in central European countries, this might be a plausible explanation, as studies on human antibodies against SINV-I showed a very low prevalence (41). Studies on antibody prevalence in regions of endemicity present 3% in Sweden and 9% in Finland (25, 32, 42). Thus, humans in Germany do not seem to become infected with SINV-I as often as humans do in areas of endemicity of Fennoscandia. Ecological studies have shown that the abundance of the most competent vector of SINV-I, Culex torrentium, is decreasing southward in Europe (43, 44). One could speculate that there is a critical abundance of Cx. torrentium that needs to be reached for an efficient-enough transmission of SINV-I in the bird population to allow spillover infections of humans. Likewise, the abundance of the bridge vector Aedes cinereus is likely of importance for the transmission of virus from birds to humans (3). Other ecological factors, such as variations in host choice by the vector and mosquito longevity, could also contribute to explain the differences in transmission. In summary, the geographical limitation of SINV fever outbreaks needs to be further investigated in studies on the pathogenicity of the different strains as well as in ecological studies on the vector and host populations in different regions.

In conclusion, our results suggest that SINV-I was successfully introduced only once into northern Europe from central Africa, probably via migratory birds. This introduction led to the establishment of endemic SINV circulation in Sweden, and from there, SINV-I spread to other parts of northern, eastern, and central Europe. The emergence of reported human cases is potentially due to synergistic effects, including awareness of the disease in the community, ecological circumstances, and undiscovered host and viral genetic factors.

## MATERIALS AND METHODS

### Virus strains.

SINV strains for the present study are listed in Table 3 and were originally isolated from a number of sources, including mosquitoes, birds, and humans (e.g., see references 10, 20, and 45; J. O. Lundström, J. Hesson, M. Schafer, O. Ostman, T. Semmler, M. Weidmann, and M. Pfeffer, unpublished data). All strains sequenced in this study were propagated in Vero cells as described previously by Hesson et al. (45) or in suckling mice as described previously (46) and stored at −80°C.

**TABLE 3:** Sindbis virus genotype I strains included in this study

Isolate	Yr of isolation	Location(s) (region)^*a*^	Isolation source^*b*^	E2 GenBank accession no.	GenBank accession no.	Reference(s)
E597	2002	Sweden (N)	Aedes rossicus		MK045224	This study
E594	2002	Sweden (N)	A. rossicus		MK045225	This study
84M140	1984	Sweden (E)	Culiseta morsitans		MK045226	This study
83M108	1983	Sweden (E)	Culex pipiens/Cx. torrentium		MK045227	This study
83M107	1983	Sweden (E)	*Cs. morsitans*		MK045228	This study
Vmork1/92	1983	Norway	*Aedes* spp.	U62467	MK045229	This study, 22
86828	1985	Sweden (E)	*Cs. morsitans*		MK045230	This study
95M116	1995	Sweden (N)	A. cinereus	FJ744539	MK045231	This study, 10
86752	1985	Sweden (S)	*Cx. pipiens/Cx. torrentium*		MK045232	This study
85M78	1985	Sweden (E)	*A. cinereus*	U62460	MK045233	This study, 22
85M94	1985	Sweden (E)	*Cx. pipiens/Cx. torrentium*	U62469	MK045234	This study, 22
E917	2002	Sweden (N)	*Cx. pipiens/Cx. torrentium*		MK045235	This study
85M68	1985	Sweden (E)	*Cs. morsitans*	U62452	MK045236	This study, 22
86520P3	1985	Sweden (S)	*Cx. pipiens/Cx. torrentium*		MK045237	This study
E945	2002	Sweden (N)	*A. rossicus*		MK045238	This study
09_M_991	2009	Sweden (N)	*Cx. torrentium*	KF297653	MK045239	This study, 45
F231	2003	Sweden (N)	*A. cinereus*		MK045240	This study
09_M_519	2009	Sweden (N)	*Cx. pipiens*	KF297652	MK045241	This study, 45
09_M_564	2009	Sweden (N)	*Cx. torrentium*	KF297646	MK045242	This study, 45
09_M_526	2009	Sweden (N)	*Cx. torrentium*	KF297644	MK045243	This study, 45
85M134	1985	Sweden (E)	*Cx. pipiens/Cx. torrentium*	U62444	MK045244	This study
09_M_358	2009	Sweden (N)	*Cx. torrentium*	KF297651	MK045245	This study, 45
SAAR_18141	1976	South Africa	Culex univittatus	U62471	MK045246	This study, 22
AR18132	1974	South Africa	*Cx. univittatus*	U62463	MK045247	This study, 22
MP684	1960	Uganda	Mansonia fuscopennata	U62480	MK045248	This study, 22
4251SM6	1969	Cameroon	Mosquito	AF339477	MK045249	This study; R. M. Kinney and M. Pfeffer, unpublished data
SAAR_6071	1964	South Africa	*Cx. univittatus*	U62479	MK045250	This study, 22
SK2_SM7V1	1972	Slovakia	Cricetus cricetus (M)	U62458	MK045251	This study, 22
AZ_16	1977	Russia, Azerbaijan	Nycticorax nycticorax (B)	U62464	MK045252	This study, 22
GREIS	1975	Italy	Hyalomma marginatum (T)	U62456	MK045253	This study, 22
M1855	1967	Israel	Cx. pipiens *molestus*	U62473	MK045254	This study, 22
R2_SM7V1	1975	Slovakia	Rana ridibunda (A)	U62459	MK045255	This study, 22
1038	1964	Israel	Streptopelia turtur (B)	U62443	MK045256	This study, 22
SA80_480	1980	Saudi Arabia	*Cx. univittatus*	FJ744536	MK045257	This study, 10
SA80_394	1980	Saudi Arabia	*Cx. univittatus*	FJ744535	MK045258	This study, 10
SA80_370	1980	Saudi Arabia	*Cx. univittatus*	FJ744534	MK045259	This study, 10
BNI_10865	2016	Germany	*Cx. pipiens*		MF589985	6
15Z03121	2015	Germany	*Cx. torrentium*		MG779533	6
LEIV_9298	1983	Russia	Mosquito		JQ771799	17
Kiihtelysvaara2002	2002	Finland	Homo sapiens		JQ771798	17
Johannes2002	2002	Finland	Homo sapiens		JQ771797	17
Edsbyn	1982	Sweden	Mosquito		M69205	58
Ilomantsi2002B	2002	Finland	Homo sapiens		JQ771795	17
Ilomantsi2002A	2002	Finland	Homo sapiens		JQ771794	17
28_9	2009	Germany	*Cx. torrentium*		GU361118	41
5_3	2009	Germany	Anopheles maculipennis		GU361116	41
Ilomantsi2002C	2002	Finland	Homo sapiens		JQ771796	17
Lovanger	2013	Sweden (L)	*Cs. morsitans*		KF737350	16
Z158523	2013	Germany	*Cx. pipiens*		MG779535	6
Berlin2010A	2010	Germany	Corvus corone *cornix* (B)		JX570540	59
Ilomantsi2005M	2005	Finland	Mosquito		JQ771793	17
NVS_305	2007	Kenya	Culex vansomereni		KY616988	60
ArB7761	1977	Central African Republic	*Cx. cinereus*		MH212167	46
HB741570	1975	Central African Republic	Homo sapiens		MF409178	V. Tricou, E. Nakoune, B. Selekon, M. Kazanji, and N. Berthet, unpublished data
Babanki	1969	Cameroon	Mosquito		HM147984	47
KSM_1008	2007	Kenya	*Culex* spp.		KY616986	60
BUD_12001	2010	Kenya	*Cx. univittatus*		KY616984	60
Girdwood_2017	1962	South Africa	Homo sapiens^*c*^		MF459683	61
Girdwood_SA	1962	South Africa	Homo sapiens^*c*^		U38304	62
YN87448	1998	China	Unknown		AF103734	G. L. Zhou, G. D. Liang, L. Li, S. H. Fu, Q. Jin, H. L. Zhang, W. L. Huang, and Y. D. Hou, unpublished data
Prototype AR399	1952	Egypt	*Cx. pipiens/Cx. univittatus*		J02363	63
S_A_AR86	1954	South Africa	*Culex* spp.		U38305	62
H7	2013	Germany	*Cx. pipiens*		MG779534	6
BONI_584	2013	Kenya	Aedes tricholabis		KY616985	60
BONI_566	2013	Kenya	A. ochraceus		KY616987	60
ArB489	1985	Central African Republic	Arthropod		MF409177	

a Letters in parentheses indicate regions within Sweden shown in Fig. 1 (N, Nedre Dalälven; E, Edsbyn; S, Sundsvall; L, Lövånger).

b Letters in parentheses indicate the isolation source M, mammal; B, bird; T, tick; A, amphibian.

c This strain was isolated from a human in 1962 and sequenced twice.

### RNA extraction and sequencing.

Total RNA of the SINV-infected cell culture supernatant was extracted using the QIAamp viral RNA minikit (Qiagen, Hilden, Germany) according to the manufacturer’s instructions. cDNA was synthesized using the RevertAid H minus first-strand cDNA synthesis kit (Thermo Scientific, Vilnius, Lithuania) in a 20-μl mixture containing 2 μl of random hexamers (10 pmol/μl), 2 μl 10 M deoxynucleoside triphosphate (dNTP), and 5 μl RNA. The presence of SINV RNA was confirmed by quantitative PCR (qPCR) as described previously (41).

PCRs, using modified oligonucleotide primers that cover the complete genome of SINV (17) (Table 4), were performed using Phusion Flash (Thermo Scientific, Lithuania). PCR amplicons from the same strain were pooled and purified using the QIAquick PCR purification kit (Qiagen, Hilden, Germany). Illumina sequencing libraries were constructed using multiplex PCR products (1 ng of input DNA) and the Nextera XT DNA library prep kit (Illumina, San Diego, CA, USA) according to the manufacturer’s instructions. Sequencing was performed on an Illumina MiSeq instrument using MiSeq reagent kit version 2 (Illumina, San Diego, CA, USA). Assembly of the sequence data was done using the CLC genome workbench, using the SINV Babanki strain (GenBank accession number HM147984) as the reference sequence (47). Low-coverage regions were closed by conventional PCR, with primers designed according to known sequences in the flanking regions.

**TABLE 4:** Primers used in this study

Oligonucleotide	Sequence	Positions^*a*^	Length of the product (bp)
SINV F1	GAATCRAACAGCCGACCAAT	25–44	641
SINV R1	GTCGGCCCAGTTAGTGTTGT	646–665	
SINV F2	ACTGGATTGGCTTTGACACC	583–602	485
SINV R2	CGGGATATACGTGCACACAG	1048–1067	
SINV F3	CAAACAATAGCGAGGGCTTC	979–998	793
SINV R3	GAGGTTGGTGAAACGACGAT	1752–1771	
SINV F4	TGGCAGACAAAGACATCGAG	1606–1625	681
SINV R4	GTGCCGTGACAGTTGACTTG	2267–2286	
SINV F5	GCGATGCGTTAAGAAGGAAG	2096–2115	1,088
SINV R5	ACTGGCAACCGGTAAGTACG	3164–3183	
SINV F6	GAGGACTGGGAAGCTGAACA	3030–3049	1,062
SINV R6	TCCRTCTCTTGTACCCTCRT	4072–4091	
SINV F7	GRCCAGAKTGYGTCTCAAGCA	3959–3978	600
SINV R7	TCGATTCGTTCCTTCCACTT	4539–4558	
SINV F8	ATCAAGTCTGTCGCCATTCC	4401–4420	1,086
SINV R8	CATGGATACCCCACCAAAAG	5467–5486	
SINV F9	TTTAGCGGATCGGACAATTC	5238–5257	1,298
SINV R9	GCGGTGACGAACTCAGTAG	6517–6535	
SINV F10	CTGGAYTCAGCGACATTCAA	6423–6442	1,127
SINV R10	TTGCTCTGGGCAAAAGTTCT	7530–7549	
SINV F11	GCCCTGCTAGATGAAACGAA	7416–7435	1,171
SINV R11	TATCGTAGGCCTCGTGGTTC	8567–8586	
SINV F12	GGATAACTCAGGTCGGGTTG	8312–8331	768
SINV R12	TTACCGTGAACGGGAGGTAG	9060–9079	
SINV F13	AGCGTGACGGTTAGCATAGC	8964–8983	909
SINV R13	CAGCGAAGTTGGAATTACGG	9853–9872	
SINV F14	TACCATCGCCATCCTGTGTA	9714–9733	949
SINV R14	CTGGTTTCATCGCTCCGTAT	10 643–10 662	
SINV F15	ACGGAGTTACACCAGGAACG	10 516–10 535	1,121
SINV R15	TATGCACCAYGCTTCCTCAG	11 617–11 636	

a The reference genome was from reference 17.

### Phylogenetic analysis.

Publicly available full-genome sequences of SINV were retrieved from the National Center for Biotechnology Information (NCBI) website (https://www.ncbi.nlm.nih.gov/). All sequences, 66 in total, were then aligned using MAFFT with default settings, followed by manual refinement using AliView (48, 49).

Several alignments were constructed based on (i) the complete open reading frames (ORFs), (ii) the single genes (nsP1 to -4, C, E1, E2, E3, and 6K/TF), (iii) complete and partial E2 sequences, and (iv) the complete ORFs excluding recombinant sequences. The best-fit evolutionary nucleotide substitution model following jModelTest analysis, GTR+F+I+G4 (general time-reversible model with empirical base frequencies, allowing for a proportion of invariable sites, and a discrete gamma model with default 4 rate categories), was used in all phylogenetic analyses (50). Potential recombination events were investigated using the Phi test in SPLITS TREE 4.0 (51), Simplot version 3.5.1 (52), and RDP3 (53). Recombination events were determined using RDP, GENECONV, Bootscan, Maxchi, Chimera, SiSscan, Phylpro, LARD, and 3Seq. In addition, alignment 4 was translated into amino acids, and amino acid positions were compared using AliView.

To reconstruct the evolutionary history of the SINV-I complex, Bayesian phylogenetic trees of the complete ORFs and separated genes (nsP1 to -4, C, E3, E2, 6K/TF, and E1) of SINV-I were constructed by employing MrBayes v.3.2.6 (54). Bayesian analysis consisted at least 5 million Bayesian Monte Carlo Markov chain (MCMC) generations sampling every 1,000 generations. The run was continued until convergence was obtained (average deviation, <0.01) and with a 25% burn-in. To further infer the evolutionary rates and divergence time of SINV-I, we first performed a regression of root-to-tip genetic distances against date of sampling by using TempEst (55). The whole data set (64 sequences) showed no clocklike structure (correlation coefficient, −0.0904). Thus, only the sequences from clades A and B (43 sequences; correlation coefficient, 0.5128) were used for the evolutionary rate estimation. We employed six different combinations of demographic and molecular clock models (S2) and ran 50 million Bayesian MCMC generations sampling every 1,000 generations, implemented in BEAST version 2.3.1 (56) (Table 5). Model comparison performed by using a marginal-likelihood estimator in two approaches, path sampling (PS) and stepping-stone sampling (SS), selected strict clock and exponential population as a better model for data analysis, with the log Bayesian factor (BF) value over at least 25. In all analyses, strain ArB489 from central Africa, isolated in 1985 (GenBank accession number MF409177), was used to root the tree. All computations were run using the CIPRES computational cluster (http://www.phylo.org/index.php/). Finally, trees were viewed and edited using FigTree v1.4.2 (http://tree.bio.ed.ac.uk/software/figtree/).

**TABLE 5:** Model comparison by BEAST analysis^*a*^

Molecular clock model	Demographic model	Mutation rate	95% HPD	Log marginal likelihood (SS)	Log marginal likelihood (PS)
Relaxed clock, exponential	Coalescent exponential population	1.045 × 10^−4^	5.6508 × 10^−5^, 1.6026 × 10^−4^	−23,632.61	−23,632.33
Relaxed clock, log normal	Coalescent exponential population	1.5723 × 10^−4^	6.6297 × 10^−5^, 2.6341 × 10^−4^	−23,632.24	−23,631.94
Relaxed clock, exponential	Coalescent Bayesian skyline	1.225 × 10^−4^	6.8056 × 10^−5^, 1.8561 × 10^−4^	−23,622.27	−23,621.7
Relaxed clock, normal	Coalescent Bayesian skyline	1.257 × 10^−4^	7.4641 × 10^−5^, 1.8208 × 10^−4^	−23,623.27	−23,623.38
Strict	Coalescent exponential population	5.4535 × 10^−5^	4.3983 × 10^−5^, 6.5605 × 10^−5^	−23,757.07	−23,756.55
Strict	Coalescent Bayesian skyline	5.476 × 10^−5^	4.2365 × 10^−5^, 6.7008 × 10^−5^	−23,746.58	−23,746.95

a 95% HPD, 95% highest posterior density; SS, stepping-stone sampling; PS, path sampling.

A number of studies have previously used partial E2 sequences for creating SINV phylogenies (10, 22, 57). To investigate whether phylogenies based on partial E2 sequences give the same results as the phylogenies based on the complete ORF, and to be able to compare more strains, we also constructed phylogenies using all strains sequenced in this study and all available partial E2 sequences from GenBank (170 in total [minimum, 313 nucleotides {nt}; maximum, 2,200 nt]) (10).

### Data availability.

All newly sequenced strains have been deposited in GenBank under accession numbers MK045224 to MK045259.
